# The function and mechanism of action of uterine microecology in pregnancy immunity and its complications

**DOI:** 10.3389/fcimb.2022.1025714

**Published:** 2023-01-04

**Authors:** Liping Shen, Weiwei Wang, Weiwei Hou, Chenfei Jiang, Yi Yuan, Liqing Hu, Anquan Shang

**Affiliations:** ^1^ Department of Obstetrics and Gynecology, Changning Maternity & Infant Health Hospital, Shanghai, China; ^2^ Department of Obstetrics and Gynecology, the Second Affiliated Hospital of Soochow University, Suzhou, China; ^3^ Department of Laboratory Medicine, The Second People's Hospital of Lianyungang & The Oncology Hospital of Lianyungang, Lianyungang, Jiangsu, P.R., China; ^4^ Department of Pathology, Tinghu People's Hospital of Yancheng City, Yancheng, Jiangsu, P.R., China; ^5^ Department of Laboratory Medicine, Shanghai Tongji Hospital, School of Medicine, Tongji University, Shanghai, China; ^6^ Department of Laboratory Medicine, Ningbo First Hospital & Ningbo Hospital of Zhejiang University, Ningbo, Zhejiang, P.R., China

**Keywords:** pregnancy immunity, vaginal microbiota, vaginal dysbiosis, uterine microflora, uterine dysbiosis

## Abstract

The human microbiota influences physiology, disease, and metabolic reproduction. The origin of uterine bacteria is controversial. The main assumption is that the germs enter the uterine cavity from the vagina through the cervical canal, bloodstream, fallopian tubes, and gynecological surgical channels. Understanding the microbiota at various anatomical sites is critical to the female reproductive system and pregnancy. Today’s study focuses on the role of uterine bacteria in pregnancy and embryo implantation. According to our findings, the uterine microbiome influences embryo implantation and pregnancy outcome. Pregnancy is a natural, evolutionarily selected approach to human reproduction. During pregnancy, the microbiota of the reproductive tract changes, facilitating the maintenance of pregnancy, and the human immune system undergoes a series of changes that recognize and adapt to the non-self. From the beginning of pregnancy, a non-self fetus must establish a placenta of embryonic origin to protect itself and promote growth; the VMB tends to be more stable and lactobacillus-dominated in late gestation than in early gestation. Any material that disrupts this connection, such as microbial changes, is associated with a higher risk of poor health and poor pregnancy outcomes in women (eclampsia). The presence of any material that disrupts this connection, such as microbial changes, is associated with a higher risk of poor health and poor pregnancy outcomes (preeclampsia, preterm birth, gestational diabetes, etc.). In this work, we review the last decade of relevant research to improve our understanding of the mechanisms by which the microbiota of the female reproductive tract influences female reproductive health. This work discusses the mechanisms associated with the reproductive tract microbiota and pregnancy immunity, as well as the impact of an abnormal microbiota on adverse pregnancy outcomes. Emphasis is placed on the characteristics and sources of the female vaginal, uterine, and placental microbiota and the importance of a well-stabilized local human microbiota and immune system for embryo implantation, placental development, fetal growth, and pregnancy outcome.

## 1 Introduction

The human microbiome consists of trillions of microbes living in various parts of the body, including bacteria, archaea, protozoa, fungi, and viruses (the collective genome is called the “microbiome”) (Lloyd-Price et al., 2016; Schirmer et al., 2018). They participate in regulating food metabolism and preserving the integrity of the intestinal epithelium. They are present in the human body as a result of their intricate interaction with numerous elements of host physiology and metabolism. In supporting the host in nutritional absorption and immunological development, they serve a crucial function in protecting the host against infections (Sepich-Poore et al., 2021). The age of multi-omics in humans has increased scientific development in all fields of biology, notably the study of the microbial population and normal flora, during the last decade. In the eighteen years following the release of the first human genome, the Human Microbiome Project (HMP) has shifted its focus from oral and intestinal cultures to the molecular profiling of microbial biochemistry in all human ecological settings (Gill et al., 2006; Costello et al., 2009). Diverse microorganisms have distinct properties in various bodily areas. For instance, higher variety in the gastrointestinal system is generally anticipated, but it may be linked with dysbiosis and an increased risk of adverse outcomes in the female reproductive tract. The Human Microbiome Project (HMP) program consists of two parts (HMP1 and HMP2). HMP2 extends the analysis of host biological characteristics and the microbiome, and a longitudinal cohort study was conducted on three typical microbiome-associated diseases: pregnancy and PTB (vaginal microbiome of pregnant women), Inflammatory Bowel Disease (microbiome), and Prediabetes (intestinal and nasal microbiome). The majority of pregnancy and PTB research has focused on the vaginal microbiome, whereas the uterine microbiome has received very less attention. Therefore, we will further explore the relevance of uterine microbiome to PTB and its research progress.

## 2 Materials and methods

### 2.1 Ethics statement

All individuals involved in this study signed informed consent. The study was approved by Ethics Committee of Shanghai Tongji Hospital and in line with the ethical standards of the Declaration of Helsinki.

### 2.2 Clinical sample collection

Intrauterine secretions were collected from 131 cases of pregnant women during pregnancy with an average age of 52.7 ± 6.25 from the Shanghai Tongji Hospital. In accordance with the diagnostic criteria of the International Federation of Gynecology and Obstetrics (FIGO) systems (2014), patients were examined by two senior or more gynecologists by combined pathogenic microbiology and other examinations. They were diagnosed as having an altered microbial abundance in the uterus in combination with pregnancy, while not having HPV infection or cervical lesions. The samples were stored at -80°C.

### 2.3 Uterine microbiome analysis using 16s rRNA gene sequencing

In order to perform uterine microbiome analysis of PTB, the V3-4 region of 16S-rRNA gene was amplified and sequenced by Guangzhou Kidio Biotechnology Co.

## 3 Results

**Figure 1:**
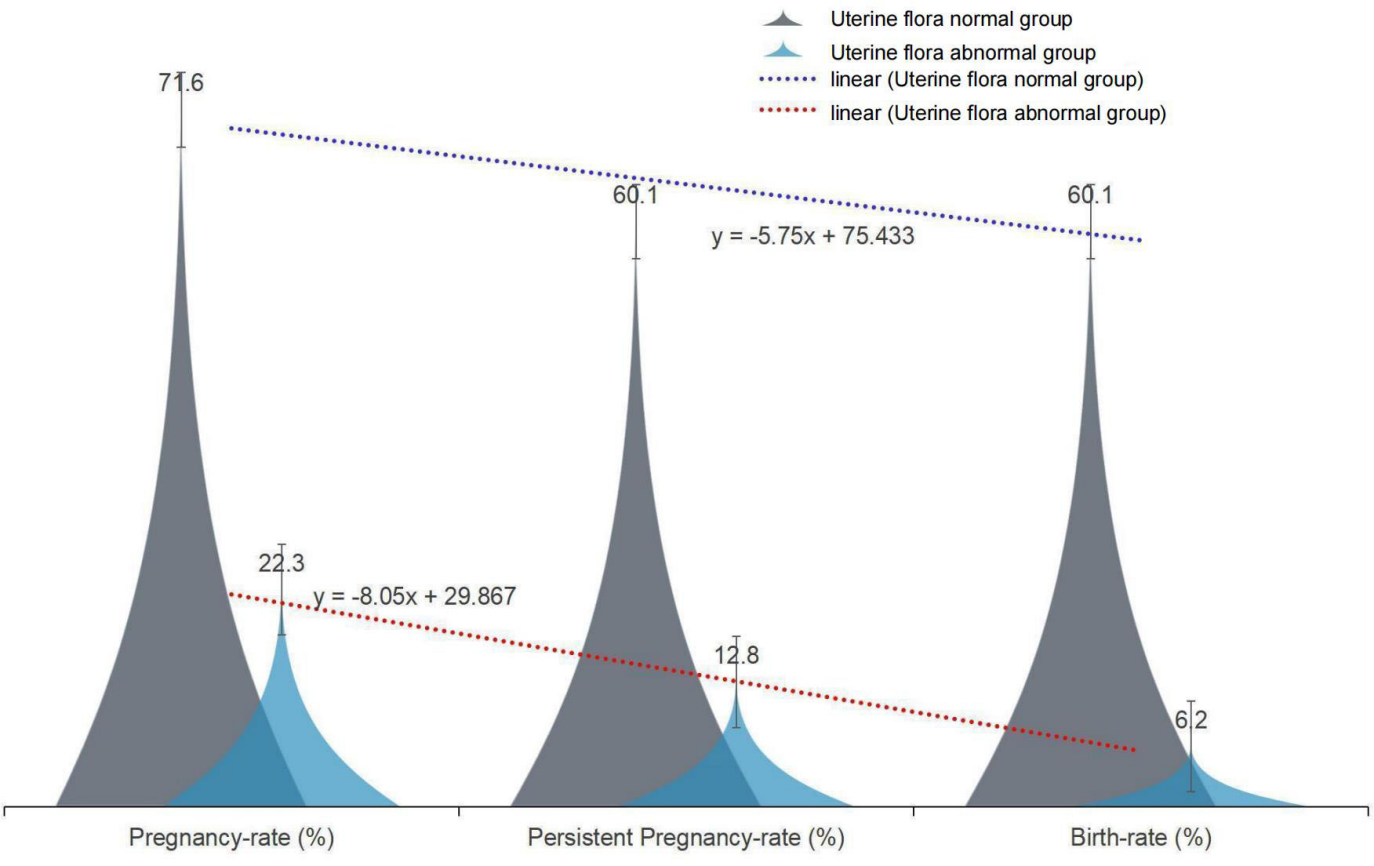
The effect of normal and abnormal uterine microbiota on pregnancy outcome.

We retrospectively analyzed the uterine secretions of 131 patients in our fertility center by 16S RNAseq and found that dysbiosis of the uterine cavity microbiota has an impact on implantation and pregnancy outcome (**Figure 1**).

## 4 Discussion

### 4.1 Vaginal microbiome

The majority of women have strains of the genus Lactobacillus that produce lactic acid, and these strains predominate in the vaginal microbiota. Other compounds generated by Lactobacillus in the vaginal milieu, such as hydrogen peroxide and bacteriocin, limit the development of potential pathogens in addition to lactic acid. These molecules are created in the vaginal microenvironment. The changes in vaginal balance in human nature lead to dysbacteriosis, abnormal inflammatory responses, and immune responses that result in the occurrence of many types of diseases of the female reproductive system. In 2011, a landmark study of high-throughput 16S rRNA taxonomic analysis classified vaginal colonization in women of non-pregnant reproductive age into five types, namely CSTI (L. crispatus), CST II (L. gasseri), CST III (L. iners), and CST V (L. jensenii), by Ravel et al. and a fifth - more complex - CST IV (composed mainly of several anaerobic species), CST IV was further subdivided into IVA (dominated by BVAB1), IVB (dominated by Gardnerella vaginalis), IVC0 (dominated by Prevotella), IVC1 (dominated by Streptococcus), IVC2 (dominated by Enterococcus), IVC3 (dominated by Bifidobacterium), and IVC4 (dominated by Bifidobacterium) (dominated by Staphylococcus) (Ravel et al., 2011). In this study, the structure of vaginal microflora was different in different ethnic groups. Studies have shown that vaginal pH often fluctuates. In some women, menstruation or sexual activity may trigger a switch between CSTs at different times (Gajer et al., 2012). Follicular phase estrogen causes vaginal epithelial cells to thicken and secrete more glycogen; this breakdown produces lactic acid and hydrogen peroxide, which lower vaginal PH and facilitate and tend to stabilize the proliferation of lactobacilli, while reducing the number and diversity of other anaerobes (Onderdonk et al., 2016). Menstrual blood neutralizes the acidic vaginal milieu, and a rise in intravaginal PH results in a substantial increase in the number of anaerobic microorganisms as symbionts, with less Lactobacillus (Kaur et al., 2020). Vaginal flora The imbalance can lead to susceptibility to infection or complications in reproduction. When vaginal microecological homeostasis is disturbed, there is an imbalance of vaginal flora, abnormal inflammatory responses and immune responses, etc. This may lead to bacterial vaginosis, intraepithelial neoplasia of the cervix, gynecologic cancers, and other gynecologic diseases. There is now mounting evidence that the vaginal microbiota influences the fetal and neonatal health of pregnant women (Ravel et al., 2011). The most common gynecologic condition in women of reproductive age is vaginal microflora, which is also associated with an increased risk of genital infections, pelvic inflammatory disease, and adverse pregnancy outcomes (including preterm delivery、preeclampsia) (Younes et al., 2018; Fettweis et al., 2019). Several studies in recent years have demonstrated the important role of the female reproductive tract microbiota in the prediction and prevention of preterm birth (**Table 1**) (Witkin et al., 2019).

**Table 1:** Latest Research on Female Reproductive Tract Microbes and Preterm Birth.

References	Patients (N)	Vaginal taxa increased in relative abundance in PTB	Vaginal taxa reduced in relative abundance in PTB
Payne et al. (2021)	premature birth (n=58)	Gardnerella spp./Lactobacillus iners/Ureaplasma spp.	Lactobacillus crispatus/Lactobacillus gasseri/Lactobacillus jensenii
Kosti et al. (2020)	premature birth (n=112) term infant (n=303)	Olsenella/Clostridium sensu stricto/Didiister/Prevotella/Megasphaera/Atobium/Gardnerella/Aerococcus	Lactobacillus app.
Fettweis et al. (2019)	premature birth (n=45) term infant (n=90)	Aerococcus spp./BVAB1/BVAB2/Coriobacteriaceae species/Dialister spp./Parvimonassp./Prevotella spp./Sneathia amnii/Sneathia sanguinegens/TM7-H1	Lactobacillus crispatus/Lactobacillus spp.
Elovitz et al. (2019)	premature birth (n=107) term infant (n=432)	Mobiluncus curtsii/Mobiluncus mulieris/Sneathia sanguinegens/Atopobium spp./Megasphaera spp./Lactobacillus iners/β-defensin-2	Lactobacillus app.
Witkin et al. (2019)	premature birth (n=56) term infant (n=284)	Ureaplasma spp.	Lactobacillus app.
Callahan et al. (2017)	premature birth (n=50) term infant (n=85)	Gardnerella spp.	Lactobacillus crispatus/Lactobacillus spp.
DiGiulio et al. (2015)	premature birth (n=15) term infant (n=49)	Gardnerella spp./Ureaplasma spp.	Lactobacillus app.

Changes in vaginal flora can lead to different pregnancy outcomes. During pregnancy, estrogen levels continue to increase, and lactobacillus plays a greater role. Some studies have shown that changes in the microbiota are associated with PTB, premature rupture of membranes, chorioamnionitis, pregnancy termination, and other adverse pregnancy outcomes (Fettweis et al., 2019). A higher *in vitro* fertilization result was linked to a lower microbiota index, as measured by examination of vaginal microflora on the day of embryo transfer. Higher levels of Liszt vaginalis were associated with a higher probability of pregnancy at ICSI (Koedooder et al., 2019). In none of these studies was the flora of the upper genital tract sampled and analyzed, so the final results are not very conclusive. Therefore, it is of great importance to improve the study of bacterial flora in the upper genital tract for the emerging study of pregnancy outcomes.

### 4.2 Uterine microbiome

Is there a real fixed microbiota in the uninfected upper genital tract? This concept has long been controversial. As early as 1900, it was agreed that a healthy uterine cavity was sterile. Later, the “germ-free uterus” hypothesis was challenged by several reports in the mid-to late 1980s, even in healthy, asymptomatic women in whom the uterus was found to be enriched with endemic bacteria using a bacterial culture. The cervical mucus suppository can inhibit, but not block, the passage of microorganisms from the vagina through the cervical canal into the uterine cavity (Hansen et al., 2014). Recently, several studies have targeted the putative endometrial microbiota by 16S rRNA sequencing, and each study has documented the presence of the uterine microbiota (Fang et al., 2016; Franasiak et al., 2016; Moreno et al., 2016; Verstraelen et al., 2016; Chen et al., 2017; Miles et al., 2017; Winters et al., 2019; Lozano et al., 2021; Oberle et al., 2021; Wang et al., 2021; Wessels et al., 2021). **Table 2** provides an overview of these recent studies over the last 5 years that rely on 16S rRNA sequencing to assess the microbial composition of the human uterus. Franasiak et al. (2016) examined the endometrial microbiota of 33 women by collecting material from catheter tips used for ET during assisted reproductive technology. Lactobacillus and Flavobacterium were identified as the most abundant genera in the nonpregnant and pregnant groups. In a study focused on the relationship between endometrial polyps and local microbiota, In 2016, another pilot study showed that pregnancy rates in women with a non-lactobacillus dominant (NLD) uterine environment dropped by nearly 40 percent after uterine microbial composition was identified as a new focus (Moreno et al., 2016). Many scientists have attempted to describe the natural microbiota of the uterus to determine what impact the uterine microbiota has on pregnancy outcomes (Chen et al., 2017).

**Table 2:** Recent advances in research on the microbial composition of the uterus.

References	Materials	Experimental grouping criteria	Uterine microbiology-related outcomes	Conclusion
Oberle et al., 2021	33 Infertile Women	long-read Nanopore sequencing; short-read sequencing	14 infertile women (60.9%) were predominantly Lactobacillus (>80% of reads were directed to Lactobacillus).	This study established a 16S rRNA gene long-read nanopore sequencing method for *in situ* analysis of the endometrial microbiome, which has the advantages of low cost and portability and can be widely used
Wang et al., 2021	145 females aged 19-71 years	Divided into 6 groups for every 10 years	Vaginal and uterine microbial diversity decreased with age in endometritis patients compared to healthy controls. Uterine microorganisms (phylum): Firmicutes, Bacteroidetes, Proteobact, Eria, Actinobacteria, Fusobacteria, Others.	Microorganisms in the uterine cavity establish a distinct ecosystem in a small area. The uterine and vaginal microbiotas differ in structure, composition, and age-related changes. With aging, vaginal and uterine microbiota merge.
Wessels et al., 2021	21 cases	Endometriosis case group (N = 12), Control group without endometriosis (N = 9)	Patients had a more diverse endometriosis endometriosis uterine cavity microbiota than controls in terms of abundance. Microbial plexus of the uterine cavity in the group of cases with endometriosis: Actinobacteria phylum, Oxalobacteraceae, Streptococcaceae families, Tepidimonas genus. Control group without endometriosis: Burkholderiaceae family, Ralstonia genus.	The endometrial microbiota is susceptible to disturbance in those with endometriosis.
Lozano et al., 2021	60 women	Endometritis group, Non-endometritis group	The α-diversity of the vagina and endometrium was higher in the endometritis group than in the non-endometritis group; The α-diversity was not significantly different; the endometritis group showed a microbiota pattern that was not dominated by Lactobacillus spp. and was dominated by Ralstonia, Gardnerella spp.	There is a clear relationship between changes in vaginal flora and chronic endometritis.
Veronika et al., 2018	80 infertility women	The groups were divided into fresh embryo transfer patient group and frozen-thawed embryo transfer patient group.	Fresh embryo transfer patients had 28 microorganisms. 20 pregnant women (40%) have Lactobacillus spp. (90%), L. jensenii (40%) and L. crispatus (35%). Twenty microorganisms were found in FET patients. 13 women (43.3%) conceived. L. jensenii (46.2%) and L. crispatus (23%) dominated the microbiota. 23.3% of frozen-thawed embryos had bifidobacteria, compared to 2% of fresh embryos.	The uterus is not sterile, and the uterine flora may include differences from the cervical flora, which is one of the predictors of successful embryo implantation.
Winters et al., 2019	25 women	hysterectomy group. Endometrial hyperplasia resection by hysterectomy group.	The bacterial profiles of the endometrium and cervix were similar, both being dominated by Bacteroides immobilis, Pseudomonas spp. bacteria, Bacillus cereus, and Corynebacterium spp. Lactobacilli are rare in the endometrium, whereas Lactobacillus predominates in the bacterial spectrum of the vagina and cervix.	Lactobacilli are relatively rare in the endometrium, and if endometrial microbiota are present, Bacillus immobilis, Pseudomonas spp. bacilli, Monascus spp. and Bacillus cuniculi are its main members.
Chen et al., 2017	110 women of childbearing age	Microbiota were systematically collected from six sites in the reproductive tract of Chinese women of childbearing age: (CL, lower third of the vagina; CU, posterior fornix; CV, cervical canal mucus; ET, endometrium; FLL and FRL, right and left fallopian tubes; PF, peritoneal fluid from the Douglas sulcus)	CL and CU samples contained Lactobacillus coelicolor, L. endophyticus, and other Lactobacillus spp. Lactobacillus was lower in CV samples than vaginal samples and varied. Lactobacillus was no longer prominent in ET samples, and Pseudomonas, Lactobacillus, and other bacteria were dominating. At FL opening, the percentage of Pseudomonas aeruginosa and Bacillus immobilis increased, with a median relative abundance of 1.69 for Lactobacillus. Lactobacillus was absent from PF samples, however the microbiota was varied and not comparable to FL samples.	Reflects the continuity of microbial communities on the female reproductive tract, suggesting a non-fertility setting. Microbiota and potential functions associated with the menstrual cycle or over-expressed in patients with endometriosis-induced adenomyosis or infertility were also identified. The study provided to discover the nature of the vaginal-uterine microbiota.
Miles et al., 2017	10 women	Total hysterectomy and bilateral tubo-ovariectomy	Endometrial flora: Lactobacillus, Actinomycetes, Corynebacterium, Staphylococcus	Endometrial samples from atrophic endometrium were negative for sequencing. The microbial communities present at each anatomical site were closely related between the samples and the patients. The thick-walled phylum Lactobacillus is a highly abundant genus of lactic acid bacteria.
Moreno et al., 2016	10 women of childbearing age	women of childbearing age	71.7% Lactobacillus, 12.6% Gardnerella, 3.7% Bifidobacterium, 3.2% Streptococcus, 0.9% Prevotella; if samples non-Lactobacillus dominated Atopodium, Clostridium, Gardnerella, Megasphaera, Parvimonas, Prevotella, Sphingomonas or Sneathia genera abundant	Stratification into Lactobacillus versus non-Lactobacillus dominated group (containing high proportion of Atopodium, Clostridium, Gardnerella, Megasphaera, Parvimonas, Prevotella, Sphingomonas or Sneathia genera).
Fang et al., 2016	Investigation of endometrial microbial colonization related to endometrial polyps	Healthy women group H (N=10), endometrial polyps (EP) (N=20), including EP group (N=10) and EP/chronic endometritis (CE) group (N=10)	Proteobacteria (73%), Firmicutes (14%) and Actinobacteria (5%) on phylum level; Enterobacter (33%), Pseudomonas (24%) and Lactobacillus (6%) on genus level in healthy cohort.	Differences in detected phyla/genera in patient versus control cohort less Enterobacter and Pseudomonas whereas more Lactobacillus than in diseased. Higher Shannon diversity in patient cohort
Verstraelen et al., 2016	Investigation of the presence of a uterine microbiome	Recurrent implantation failure(N=11), Recurrent pregnancy loss(N=7)	Bacteroidetes phylum, making up one-third of overall population, second most abundant: Proteobacteria (incl. Pelomonas, Beta-and Gammaproteobacteria related to Escherichia/Shigella)	High similarity in 90% of women of 75% (25% Bray–Curtis dissimilarity). Additional high abundance of Lactobacillus iners, Prevotellaamnii or L.crispatus in five women.
Franasiak et al., 2016	Characterization endometrial microbiome at the time of embryo transfer by reproductive outcome	Undergoing ART(N=33)	Lactobacillus and Flavobacterium	No association in Lactobacillus content and pregnancy outcome

DNA kits have their own unique microbiome called the “dragon group” (de Goffau et al., 2018). Researchers have indicated that low levels of kit contamination are not detected in high biomass sites, such as the intestine, while when examining extremely low biomass sites, such as the placenta or uterus, results can be confounded by the kit microbiome (Kim et al., 2017). There are studies both for and against microbial colonization of the upper genital tract, and more recently, there are studies suggesting that the uterus lacks its own microbiome (Perez-Muñoz et al., 2017). In 2019, Theis KR (Theis et al., 2019)performed multiple microbiological examinations in a study of the microbiota of cesarean placenta specimens and found no normal microbiota; the results of another study also demonstrated no microbiota in prenatal fetal feces (Kennedy et al., 2021). However, it is known that pathogenic microorganisms can cause infections of the placenta (Payne and Bayatibojakhi, 2014), uterus, fallopian tubes (Lenz and Dillard, 2018), and ovaries (Brunham et al., 2015), which affect female reproductive health and pregnancy outcomes. Therefore, further studies are needed to confirm whether the native microbiome is indeed present in the upper female reproductive tract and how it is distributed.

**Figure 2:**
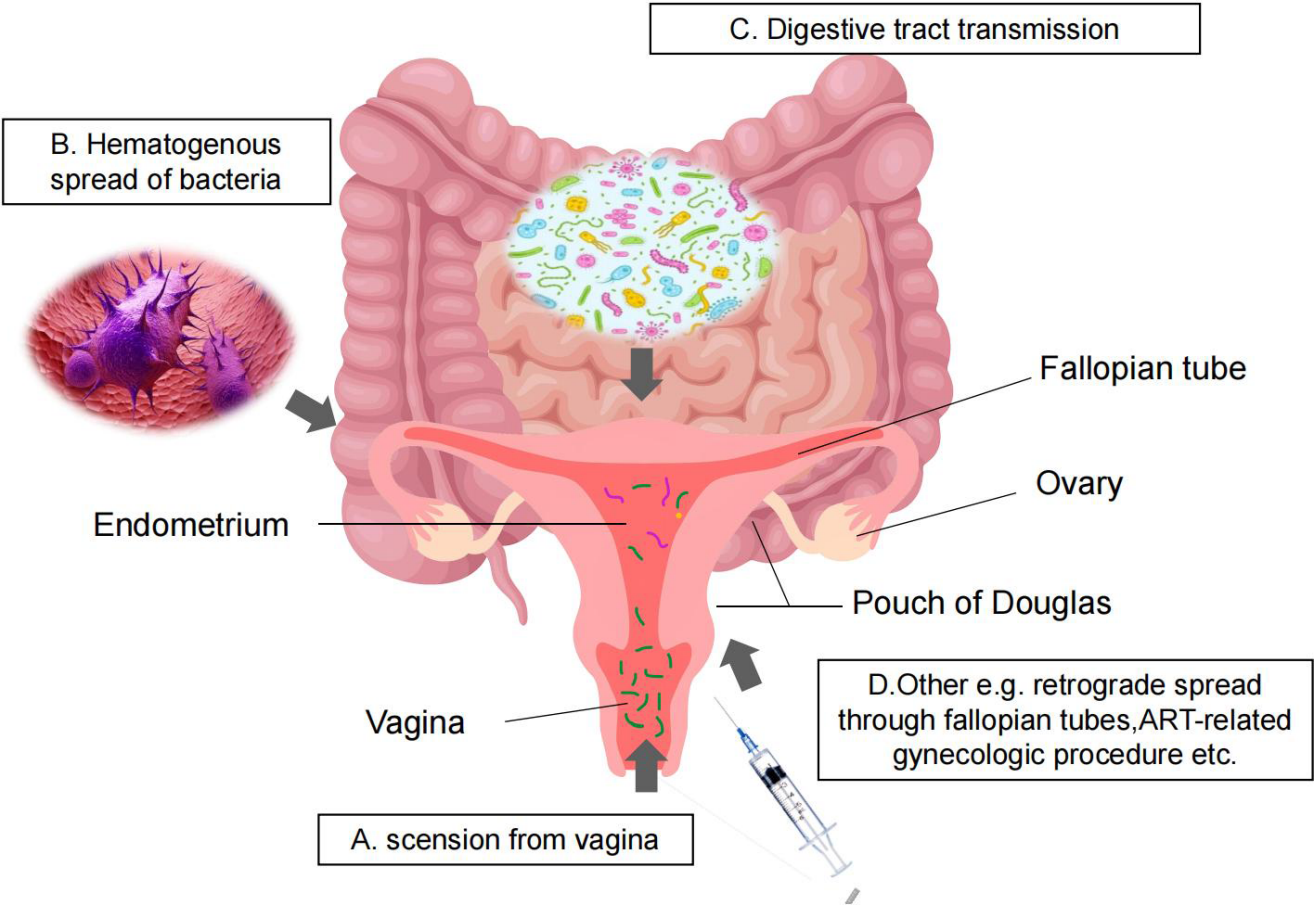
Womb microbiota transmission pathways. **(A)** scension from vagina; **(B)** Hematogenous spread of bacteria; **(C)** Digestive tract transmission; **(D)** Other e.g. retrograde spread through fallopian tubes,ART-related gynecologic procedure etc.

How can one account for the origins of the microbiome found in the uterus? Several hypotheses have been put forward to explain this. One popular idea proposes that bacterial vaginal infections may spread to the uterus if they go up the cervical canal (**Figure 2**). While there is a cervical mucus plug that blocks the transmission of bacteria to protect the uterine environment, we know that during sex, semen can enter the uterus through the cervical mucus in a small channel. Studies have shown that uterine pumps can deliver radioactive isotopes from the vagina to the uterus within 15 minutes of intercourse. Consistent with this hypothesis, a study in pregnant mice observed that bioluminescent E. coli ascended from the vagina into the uterine cavity and resulted in preterm delivery (Suff et al., 2018). However, there are significant anatomical and physiological differences between the female reproductive tract of mice and that of humans, making it difficult to generalize this observation to the human population.

Second, the possible routes of transmission are presumed blood-based transmission of intestinal and oral microbiota or other bacteria produced by the bloodstream and transmission by retrograde peristalsis of the fallopian tube into the uterine cavity. Periodontitis has been identified as a risk factor for preterm birth, and similar microbial colonization in the mouth has been found in preterm infants (Dörtbudak et al., 2005). Another study showed that a genetically labeled Enterococcus faecalis introduced into the oral cavity of mice could also be detected in the placenta (Jiménez et al., 2008). Other routes of transmission include gynecologic assisted reproductive technology procedures, such as oocyte retrieval, which can introduce vaginal bacteria into the uterus (Pereira et al., 2016); insertion or removal of intrauterine devices can introduce bacteria into the uterus (Sparks et al., 1981). Given these possible origins of the uterine microbiota, it is important to understand the microbiota at different anatomical sites for the female reproductive system and pregnancy outcomes.

### 4.3 Placental microbiome

Numerous investigations have pointed to bacterial infections as the root cause of PPROM and PTB as their respective causes. Prior to the year 2014, it was generally believed that the placenta did not have its own unique microbiome. On the placental side, an inflammatory condition known as chorioamnionitis may occur. The bacteria Bacteroides, E. coli, Gardnerella vaginalis, Mycoplasma hominis, Streptococcus, and Ureaplasma urealyticum are the most prevalent types of infectious agents. This lends credence to the hypothesis that pathogenic bacteria are capable of penetrating the amniotic and choronic membranes of the placenta (Shane et al., 2017). The idea that the placenta contains its own healthy microbiome first emerged after the groundbreaking publication by Aagaard et al. in 2014 (Aagaard et al., 2014). The results show that the placenta contains a microbiome with low abundance but high metabolic content. The placental microbiota is mainly composed of non-pathogenic commensal microbiota of Firmicutes, Ascomycetes, Proteobacteria, Bacteroidetes, and Fusobacteria. About 100 recent studies that have begun to examine the placental microbiome and its macrogenetic data have all found that it has a unique microbiota. Another recent study found that the uterine microbiota may help regulate the local immune system in preparation for embryonic implantation and placental formation (Benner et al., 2018), which could directly impact the development of PE. So how do bacteria enter the placenta? The currently proposed hypothesis is that colonization of the placenta occurs by three possible routes: first, by upstream migration from the vagina (Goldenberg et al., 2008), second, by hematogenous spread from the intestine (Perez et al., 2007), and finally, by hematogenous spread from the oral cavity (Fardini et al., 2010). However, several recent studies have challenged the idea of a neonatal and placental microbiota. Perez-Munoz et al. (Perez et al., 2007) argued that next-generation sequencing methods are limited by biomass, infection, and kit infection, and the accuracy of the measured data is questionable, so they do not support the presence of a microbiota in a healthy placental environment. Lauder et al. (2016) used PCR and Illumina sequencing to compare a series of contaminated controls to healthy placental samples and found that placental samples contained low and indistinguishable 16S rRNA copy numbers compared to extracted blanks using two different DNA extraction methods. There was also a follow-up study by Leiby et al. (2018) that examined placentas from spontaneous preterm births and found no difference between negative background controls and placenta samples. A recent groundbreaking study demonstrated that the resident microbiota in the placenta cannot be identified. This was found after researchers carefully screened for possible contaminants through a series of tests, including culture, qPCR, 16S rRNA gene sequencing, and shotgun metagenomics. The study was published in the journal Science. Even though this study was done with the most advanced technology available at the time, it is almost impossible that there is no link between the placental microbiota and the uterine microbiota if the uterine microbiota is present, as a large number of studies have shown. It is conceivable that the placental microbiota cannot be detected at present because of the very low biomass or the inadequacy of the technology available today. However, it is too early to say that there is no way to do so.

### 4.4 Microbiology and pregnancy

Pregnancy is a normal physiological process in which the female body undergoes tremendous metabolic, immunological, and endocrine changes as it enters the reproductive phase (Soma-Pillay et al., 2016). At the same time, the structure, composition, and abundance of microbial communities that colonize different parts of the body change. In early pregnancy, a completely new microbiota (Atopobium, Aerococcus, Gemella, Sneathia, Parvimonas, Gardnerella, and Megasphaera) is observed in the vaginal microenvironment, with a marked increase in the diversity of the vaginal flora and a relative decrease in Lactobacillus abundance (Kaur et al., 2020). As pregnancy progresses, estrogen levels gradually increase during mid-pregnancy and stabilize in late pregnancy. Estrogen promotes vaginal epithelial thickening and intracellular glycogen production, which can be hydrolyzed into glucose and maltose by host α-amylase, and Lactobacillus ferments glucose and maltose into lactic acid, supporting the value-added production of members of the genus Lactobacillus, and since then, Lactobacillus has become an important colony in the reproductive tract in mid- and late pregnancy. Since then, Lactobacillus has become an important colony in the reproductive tract in mid and late pregnancy and provides microenvironmental stability of the reproductive tract throughout pregnancy (Hickey et al., 2012). It has been found that the composition of the reproductive tract microbiota and its diversity are important predictors of pregnancy outcome. The diversity of vaginal microbiota is significantly higher in women with preterm delivery than in women with full-term pregnancy, and variations in “species level” (especially in Lactobacillus) are also closely associated with preterm delivery, such as Stimia, TM7-H1, and BVA-B1 (bacteria associated with bacterial vaginosis) (Fettweis et al., 2019). The dynamic composition of the microbiome and functional differences are currently hot topics in preterm birth research. Finally, maternal estrogen levels decrease rapidly at the time of delivery of the fetal placenta. The stability of the VMB is also disrupted by the mixing of fetus, amniotic fluid, placenta, and blood in the vagina, and studies have found that it can take several months for the vaginal VMB to return to a steady state, even in asymptomatic women. There is research to suggest that pregnancy intervals less than 1 year apart are associated with an increased risk of pregnancy complications (preterm births, etc.) (Shachar et al., 2016; Kangatharan et al., 2017)。

### 4.5 Immune response during pregnancy

Pregnancy is a battle against the immune system for human reproduction, because a fetus (which is not derived from a different gene from either parent) must be maintained throughout the gestation period. Like many other living things, mammals use the placenta, which develops from the embryo, to protect and nourish the developing fetus. Fertilization in the fallopian tube sets in motion a complex series of events that must be completed before the embryo can successfully implant in the uterine lining. There are three crucial phases in the implantation of a fertilized egg: the *in situ* phase, attachment (adhesion), and penetration (invasion). The immune system plays a critical role at each stage of embryonic development by generating a healthy, viable fetal egg as a mother cell that carries the maternal antigen, which is recognized by the immune system as “self.” When a sperm reaches an egg, it penetrates the fallopian tube ampulla and sets off a cascade of events leading to the development of maternal and paternal antigens. In the early stages of development, the embryo is considered a maternal cell before it begins to present its own foreign antigen. The embryo is protected from the mother’s immune cells by the zona pellucida until it is ready to transfer its antigen. In addition, certain maternal cumulus cells may provide protection during the early stages of life. After the first few days, the embryo must make contact with the maternal system. When a donor embryo is successfully implanted, the embryo must communicate to prevent attack by the maternal immune system. This communication occurs 4-5 days after embryo transfer. During this period, a series of events take place between the offspring and the mother, such as implantation into the endometrium, immune tolerance, and finally implantation (Bardos et al., 2019). We speculated that the mechanism of the uterine microbiome in human pregnancy immunity might be as follows (Bardos et al., 2019): (1) the local microbiome may affect pregnancy with clinical complications. It could be that the microbiome causes alterations in regional signaling pathways. An altered microbiome may trigger aberrant inflammatory responses *via* uterine Toll-like receptors, which may alter the cytokine milieu, altering local responses to pro-inflammatory and anti-tolerant immune cell responses. (2) It may alter the integrity of the epithelial barrier of the endometrium. Some pathogens cause a local decrease in matrix-degrading proteins, which may affect the placenta. (3) The local human microbiome has a competitive advantage. If it adapts and becomes the best nutrient scavenger in the region, it can eliminate potential invasive species through competitive exclusion. (4) Local microorganisms excrete certain metabolites, such as short-chain fatty acids, which inhibit the growth of certain species. Multiple modalities exist that would be used to selectively circumvent or engage different aspects of the immune response. The microbiota of the uterus may potentially regulate the immune cell subsets required for implantation and have implications for histomorphology (Benner et al., 2018).

Immune cells in the female genital tract play a contradictory role, both maintaining endogenous immunity to infection and creating immunological tolerance to sperm and embryo/fetus in the upper vaginal tract. Congenital lymphocytes (ILCs) are the largest population of maternal leukocytes that accumulate at the maternal-fetal interface and contribute to decidualization and implantation; NK cells are known to arise from a subset of the various ILC developmental pathways. Decidual natural killer (DNK) cells, unlike circulating NNK cells, release a variety of growth factors, angiogenic factors, and cytokines. In early pregnancy, decidual basal cells (site of implantation and trophoblast invasion/transformation) and decidual natural killer cells (DNK) make up the majority of immune cells (70%), followed by decidual macrophages (20–25%) and T cells (3–10%) (Liu et al., 2017). It is generally accepted that decidual natural killer cells (DNK) and regulatory T cells (Treg) play a critical role in promoting fetal angiogenesis, trophoblast migration, and immunological tolerance. The imbalance of immune cells in the endometrium/decidua is closely related to infertility, miscarriage, and other obstetric complications (Lee et al., 2015). The endometrium is composed of a number of immune cells, such as mast cells, macrophages, neutrophils, dendritic cells, T cells, and B cells. The composition of immune cells and cytokines at the maternal-fetal interface is in a dynamic state. Increased production of inflammatory cytokines and accumulation of decidual immune cells define the “pro-inflammatory” milieu of early pregnancy, which is essential for embryo implantation and placental development. The “anti-inflammatory phenotype” of the maternal-fetal interface develops between weeks 14 and 26 of gestation. Tolerance and maintenance of normal fetal growth depend on the development of M2-type macrophages, natural killer (NK) cells, and regulatory T cells (Tregs) in the deciduum (Yang et al., 2019).

### 4.6 NK cells in the uterus

**Figure 3:**
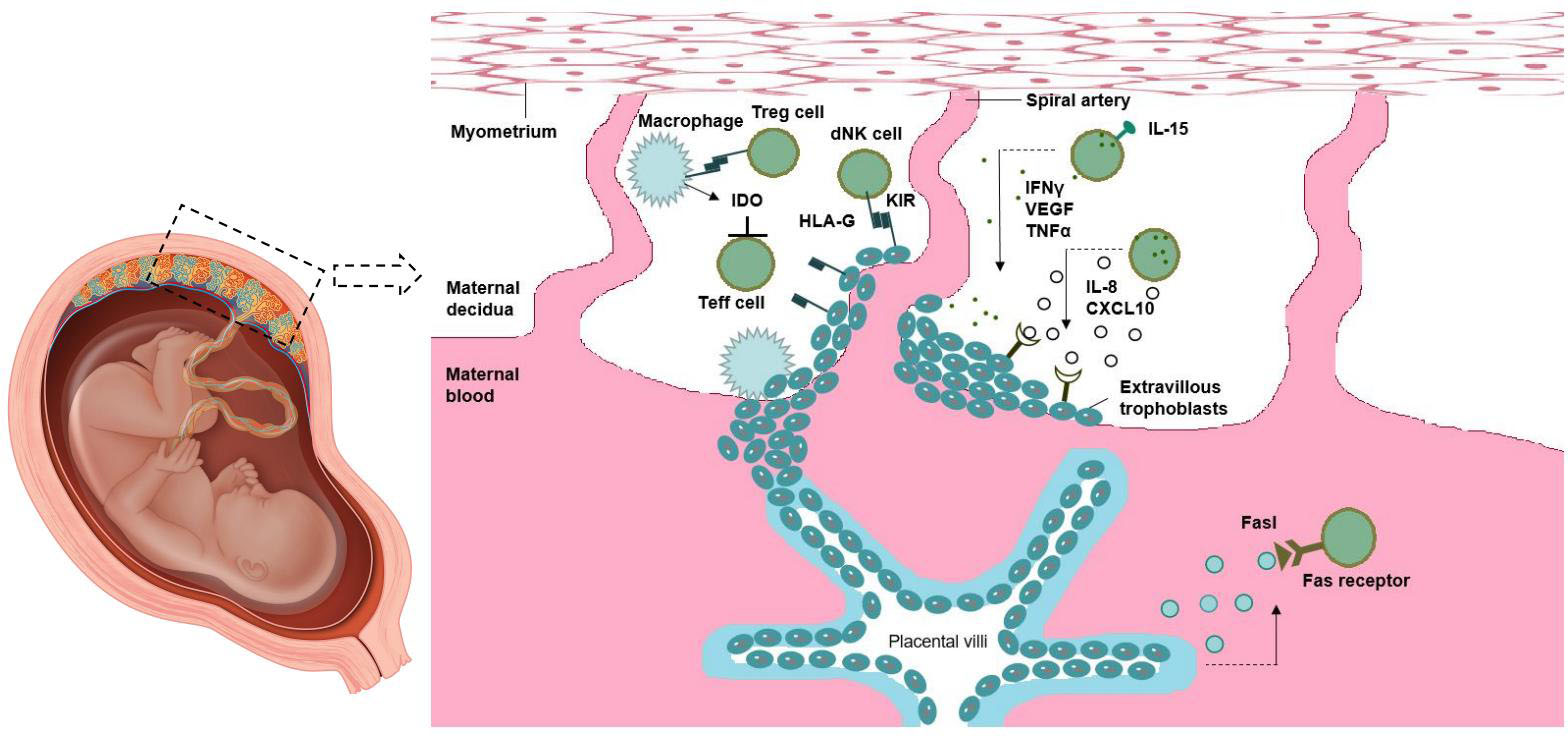
Maternal-fetal immune tolerance.

NK cells are the most abundant cells in maternal-fetal immune environment, accounting for 70% of decidua leukocytes in early pregnancy. CD56 bright CD16 phenotype was found in most of the NK cells in decidua. There are three main theories about the origin of decidual NK (DNK) cells: 1 Carlino et al. suggest that DNK cells mainly come from the recruitment of PNK cells under the action of Chemokine receptor in the uterus; And this process depends on progesterone (Carlino et al., 2008). 2 vacca et al. suggested that the interaction of various molecules in the maternal-fetal environment may induce CD34^+^ precursor cells in decidua to differentiate into DNK cells (Vacca et al., 2011). 3manaster et al. suggest that DNK cells are immature NK cells in the endometrium, which are activated by Il-15. The origin of DNK cells may be the result of the combined action of multiple mechanisms, which participate in the maintenance of pregnancy (Manaster et al., 2008). Numerous growth factors, angiogenic factors, and cytokines can be produced by DNK cells. Substances such as interferon-gamma, vascular endothelial growth factor, and tumor necrosis factor are secreted by mature DNK cells to support decidual remodeling and implantation of blastocysts. DNK also supported EVT invasion by releasing IL -8 and CXCL10. Binding of HLA-G (expressed on EVT) to the inhibitory receptor KIR2DL4 regulates DNK cell cytotoxicity. Maternal intolerance can be avoided thanks to DM phagocytizing apoptotic trophoblast cells and generating Ido, which inhibits T cell activation. To some extent, Treg cells control how active APC and effector T cells are. Exosomes secreted by syncytiotrophoblasts produce TRAIL and FASL, and the lack of MHC expression by this cell type also helps promote maternal tolerance. These cytokines promote maternal blood supply to the implantation site, stimulate trophoblast invasion, and help remodel the decidua and spiral arteries (Hanna et al., 2006). In a mouse model, interferon-γ (IFN-γ) secreted by DNK induces dilation of spiral arteries (Ashkar and Croy, 1999). The deletion of DNK cells during mouse pregnancy is related to the decrease of fetal viability and the abnormal formation of decidual structure and spiral artery at the site of implantation (Hofmann et al., 2014). Similarly, endometrial biopsies found significantly fewer NK cells in patients with unexplained infertility than in fertile women (Klentzeris et al., 1994). Interestingly, DNK cells exhibiting enhanced IFN-γ and VEGF α production by subcohorte were recently identified to be highly enriched in women with multiple births. These pregnancy-associated DNK (PTdNK) cells are transcriptionally distinct from other DNK cells and have higher expression of genes associated with NK cell activation, growth factors, and immunomodulatory proteins (Gamliel et al., 2018) (**Figure 3**).

### 4.7 T cells in the uterus

**Figure 4:**
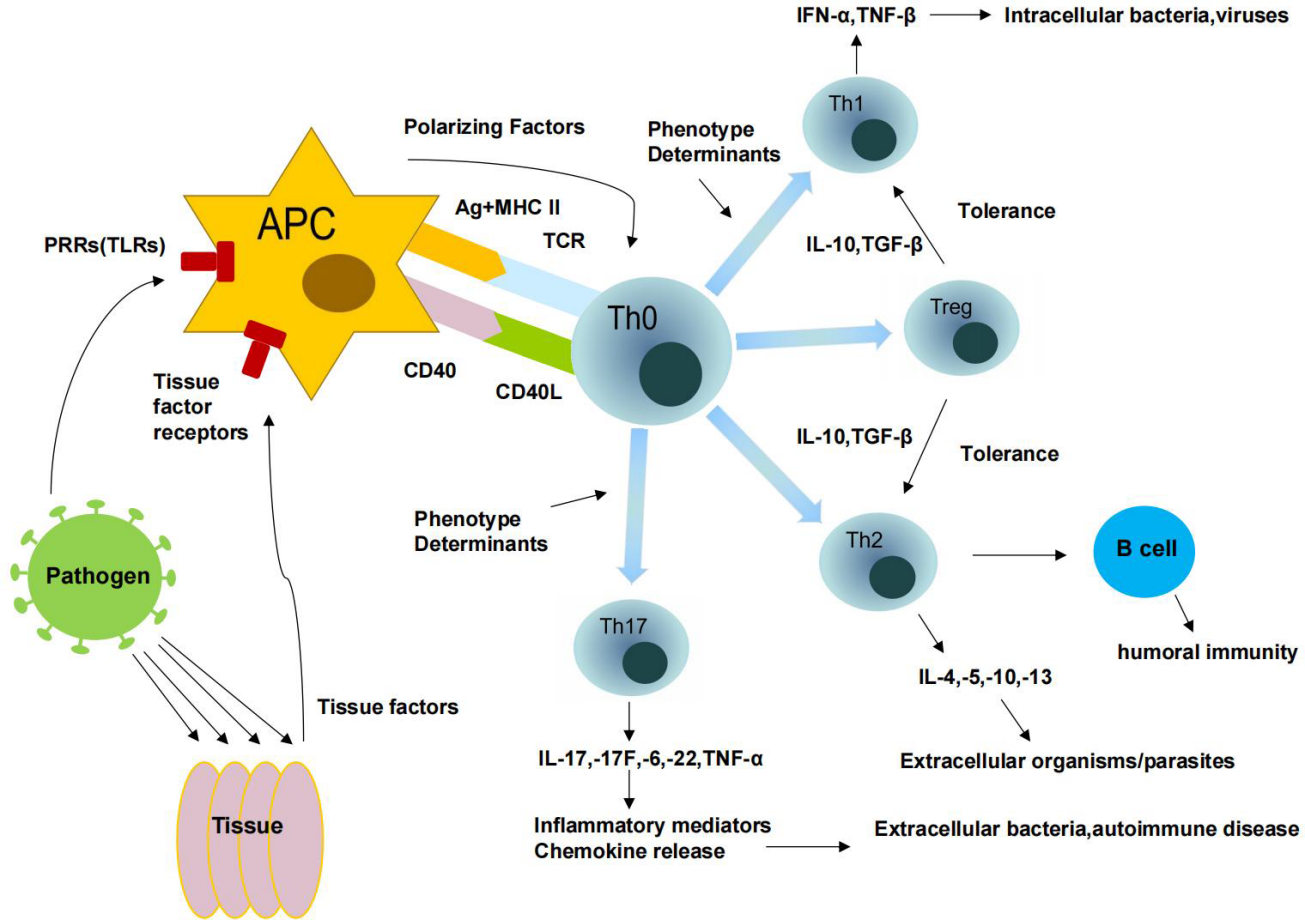
Immune response of T cells.

The various different types of T cells are part of the adaptive immune response. T helper cells (Th) are activated when they bind to T cell receptors (TCRs) to recognize peptide antigens of the major histocompatibility complex (MHC) class II presented on antigen-presenting cells (APCs). When stimulated, Th cells proliferate and/or form effector cell clones that are individually highly specialized for a particular MHC-II antigen class. These CD4^+^ Th effector cells can be divided into four distinct subsets. The different types have different functional properties based on the cytokines they secrete. Based on their phenotype, these cells are classified as either type 1 T helper cells (Th1), type 2 T helper cells (Th2), type 17 T helper cells (Th17), or regulatory T helper cells (Treg) (Zhou et al., 2009). Interferon and tumor necrosis factor are two cytokines produced by Th1 cells. Because of their ability to eliminate cancer cells and protect against intracellular infection by viruses, bacteria, and microorganisms that develop in macrophages, these cells have found wide application. Interleukin (IL)-4, IL-5, IL-10, and IL-13 are secreted by Th2 cells; these cytokines stimulate antibody formation and help fight parasites. IL-4, IL-5-induced eosinophilic granulocytes, and IL-3 and IL-4-stimulated mast cell proliferation and degranulation make parasites vulnerable, while Th2 cells activate B cells that can resist immunoglobulin (IG) production. A third group, called Th17 cells, plays a critical role in tissue inflammation and neutrophil activation to fight extracellular pathogens by secreting IL-17, IL-17F, IL-6, IL-22, and TNF-a.The triggering of immune-mediated inflammatory tolerance and negative regulation is largely dependent on their presence. By and large, Th1 cells facilitate cellular immune responses, whereas TH2 cells enhance humoral responses. Cytokines released by Th1, Th2, and Th17 populations compete with each other, and at any given time, the response of one subtype to a particular pathogen is more pronounced than that of the others (Kaiko et al., 2008). The local microbiota can influence TGF-β and increase Th17 or Treg cell populations (Brown et al., 2019). Treg cell dysfunction is associated with autoimmunity, inflammation, allergy, and acute and chronic infections (**Figure 4**).

How can an embryo convince the mother to accept itself? Treg cells have been found to acquire “non-self” antigens presented to them by downregulating Th1 and Th17 responses and promoting active tolerance to these antigens (Shevach, 2002). Treg cells inhibit the additional proliferation of T cells and the production of proinflammatory cytokines by producing TGF-β and IL10. They also suppress dendritic cell and macrophage development and activation, as well as B cell proliferation, antibody formation, and NK cell cytotoxicity (Ghiringhelli et al., 2005).As early as possible after conception, a critical period for the survival of developing embryos, Treg cells are attracted to the uterus. This is the critical period for the embryo to interact directly with the maternal immune system, such as during orthotopia, implantation, and early placental morphogenesis. Poor immune tolerance can lead to inadequate implantation, which can be the cause of intrauterine growth retardation and pregnancy termination (Maltepe et al., 2010). In addition, PEC, a common condition in late pregnancy, is associated with difficulties in placental development. A shift in transmission toward Th1 cells and away from Treg cells may be induced by the local microbiome of the intima. For example, bacterioides are thought to increase the number of Th1 cells in the body. The upregulation of Th1 cells leads to an enhancement of the local immune response and causes more local inflammation. As mentioned earlier, implantation and pregnancy outcome could be affected by increased inflammation of the endometrium. However, a healthy microbiome locally in the uterus can boost Treg cells, dampen the immune response, and induce pregnancy-or species-specific tolerance (Baker et al., 2018), thus maintaining a healthy pregnancy state and outcome.

### 4.8 Uterine microbiome and pregnancy complications

#### 4.8.1 Preeclampsia

After 20 weeks of gestation, preeclampsia (PEC) may develop, a multisystemic complication of pregnancy defined by new-onset hypertension with proteinuria or systemic disease primarily affecting the liver and kidneys. PEC currently affects 2 - 8% of global pregnancies (Jeyabalan, 2013). PEC has a complex etiology involving both maternal and fetal influences. However, the entire mechanism has not yet been deciphered. Insufficient tolerance of immune adaptations between maternal, paternal, and fetal tissues; implantation of the placenta with abnormal trophoblast infiltration; oxidative stress leading to endothelial cell dysfunction; and genetic factors, including susceptibility genes and epigenetic effects, are currently the four most likely explanations for the development of PEC. Impaired vascular function and activation of systemic inflammation in numerous maternal organs define this condition, which can be exacerbated by a number of circumstances, including infection. One of the main causes of placental dysfunction is a problem with the blood vessels in the uterus (Chaiworapongsa et al., 2014). Normal female pregnancy is associated with many hormonal, metabolic, and immunologic changes, and alterations in any of these factors may be associated with other conditions outside pregnancy, such as preeclampsia. The pathogenesis of PEC has been studied by many scientists in a variety of ways (Maynard et al., 2003; Levine et al., 2004; James et al., 2010; Verlohren et al., 2012; Souders et al., 2015; Widmer et al., 2015; DiGiulio et al., 2015; Agrawal et al., 2019; Umapathy et al., 2020). The influence of microorganisms on the multifactorial pathway of PEC has also been the focus of recent scientific studies. Microorganisms and their derivatives can trigger infections and inflammatory responses through the production of antigens and other inflammatory factors. Bacterial communities in the oral cavity, intestine, vagina, cervix, and uterus of the mother, as well as microbial communities in the placenta and amniotic fluid, may be involved in the development of PEC. A meta-analysis of epidemiological studies has shown that any viral or bacterial infection is associated with a higher risk of PEC (Rustveld et al., 2008). Another meta-analysis found that both periodontal disease and urinary tract infections during pregnancy increased the risk of PEC (Conde-Agudelo et al., 2008). Polymerase chain reaction (PCR) and next-generation sequencing showed that 12.7 percent of placental samples from women with PEC were PCR-positive for the 16S rRNA gene. Listeria monocytogenes, Salmonella, and Escherichia coli were just some of the commensal and pathogenic bacteria revealed by the samples’ microbiome (Amarasekara et al., 2015). The original theory was that an overgrowth of Bacillus or thick-walled bacteria might contribute to the development of maternal obesity. A common consequence of maternal obesity is ecological dysbiosis of the gut and “leaky gut syndrome” (Fändriks, 2017; Taddei et al., 2018). Leaky gut syndrome is defined as a weakening or failure of the epithelial barrier of the gut and is caused by stress, chronic inflammation, and dysbiosis of the gut flora. This can lead to an exacerbation of the inflammatory state during pregnancy, resulting in abnormal placental development and PEC. Maternal obesity has been shown to be an important predictor of childhood obesity and metabolic complications in the offspring. Although these mechanisms have not been fully elucidated, researchers suggest that the microbiome alters metabolism during pregnancy and modulates weight gain in both the mother and offspring (Gohir et al., 2015). On the other hand, studies have shown that long-term intake of milk-based probiotics appears to be beneficial in reducing the incidence of PEC, as they may alter the inflammatory basis of severe PEC (Nordqvist et al., 2018). Overall, these findings support the idea that microbial interactions play a role in the development of PEC and demonstrate that bacteria may contribute to inflammatory responses. Therefore, the study of the influence of bacteria on the multifactorial pathway of PEC has been a hot topic of scientific research in recent years. It has been suggested that the “gut-placenta” axis may play an important role in the etiology of PEC and that dysbiosis of gut ecology may cause the development of preeclampsia and affect the own blood pressure of women with PEC (Chen et al., 2020). We believe that a possible mechanism of pathogenesis may be that translocation of bacteria or their components to the intrauterine environment may promote inflammatory and abnormal immune responses in the placenta and maternal circulation (Chen and Gur, 2019). Although the underlying mechanisms leading to alteration of the microbiota are not known, changes in the mucosal surface of the immune system can alter the microbiota (Dunn et al., 2019). However, the specific microorganisms that can trigger PEC are still controversial. The different composition and aggregate abundance of the above maternal microbiota may contribute to impaired placental trophoblast function, leading to endothelial dysfunction and oxidative damage to nutrients and the placenta, ultimately resulting in increased maternal blood pressure, proteinuria, and PEC development (Amarasekara et al., 2015)。

#### 4.8.2 Preterm birth

Preterm birth (PTB) is a birth of less than 37 weeks of gestation due to multiple causes, including medically induced and spontaneous preterm births.PTB is the most common cause of neonatal morbidity and mortality worldwide (17.7%) (Perin et al., 2022). It is estimated that up to 25-40% of spontaneous preterm births are caused by microorganisms, and intrauterine infections caused by microorganisms can activate the innate immune system, thus increasing the risk of spontaneous preterm birth score (Goldenberg et al., 2008; Callahan et al., 2017). On the maternal mucosal surface, microorganisms secrete A2 phospholipase, which is converted into prostaglandins (PG, PGE2, PGF2A) and secretes endotoxins that stimulate a cascade of pro-inflammatory cytokines at the maternal-fetal interface leading to degradation of the collagen matrix of the cervix, fetal membranes, placenta and uterus triggering preterm labor (Yang et al., 2015). We know that with increasing weeks of gestation, the vaginal microbiome is generally dominated by Lactobacillus as the dominant flora in mid- to late pregnancy (Serrano et al., 2019). Several studies have detected Ureaplasma, Gardnerella, Clostridium sensu, Aerococcus, BVAB1 and BVAB2 flora in intrauterine infections and can induce spontaneous PTB (**Table 1**) **(** Kosti et al., 2020). Although we are not sure whether vaginal microorganisms cause intrauterine infections through upstream transmission, increased relative abundance of several microorganisms, for example, Ureaplasma and certain BV-associated taxa, were authentically detected in the VBM of women with preterm labor. Thus, it was hypothesized that microbial pathogens may enter the uterine cavity through the four aforementioned pathways, thus leading to uterine infections inducing preterm labor. We found that many PTB-associated flora are components of CST IV, such as Gardnerella, Atopobium, Dialister, Prevotella, and Sneathia. It is believed that CST IV is positively correlated with PTB. Despite the overwhelming evidence supporting the role of vaginal flora in PTB, the mechanism remains poorly understood. Several groups of studies have reported an association between the absence of Lactobacillus or poor vaginal flora and local inflammation at the cervicovaginal interface in the context of PTB, such as the analysis of cervicovaginal cytokines, chemokines and β-defensins (Fettweis et al., 2019; Elovitz et al., 2019; Florova et al., 2021). The role of the complement system in microbially driven PTB was recently proposed by Chan D in February 2022, who found that a decrease in vaginal Lactobacillus species and elevated bacterial diversity resulted in increased mannose-binding lectin (MBL), specific bacterial DNA, IgM, IgG, C3b, C5, IL-8, IL-6 and IL-1β and increased the risk of PTB (Payne et al., 2021; Chan et al., 2022). Various parameters such as cervical length (Gudicha et al., 2021), VBM composition (**Table 1**), and local expression of inflammatory cytokines (Wei et al., 2010; Fettweis et al., 2019)have been reported nowadays to predict PTB.In conclusion, microorganisms play an important role in the study of prevention of preterm birth in pregnancy.

#### 4.8.3 Gestational diabetes mellitus

The metabolic requirements of the developing fetus are met by the usual physiological adaptations of pregnancy. Gestational diabetes mellitus (GDM) is characterized by hyperglycemia and carbohydrate intolerance throughout pregnancy. Up to fourteen percent of pregnant women develop gestational diabetes, a distinct group of metabolic diseases (Szmuilowicz et al., 2019). There is already evidence of a link between microbiome and metabolism. The researchers speculate that microbiome may affect pregnancy metabolism and the development of GDM (Kuang et al., 2017). The microbes that colonize the gut affect nutrient, hunger, satiety, and lipid and glucose metabolism. Researchers examined the microbiome associated with gestational diabetes (Crusell et al., 2018). In different studies, it was found that patients with GDM had a higher frequency of microecological dysbiosis and a lower overall microbial diversity. More pathogens, including more viral and fungal species, were found in patients with GDM (Wang et al., 2018). We hope that the relationship between microecological disorder and gestational diabetes mellitus needs further study in the future.

### 4.9 Effect of microbiome on human immunity and implications for pregnancy

It is a new concept that the microbial community can influence the function of the host immune system and that the host immune system and the microbial community maintain a symbiotic relationship. Bacteria in other mucous membranes, such as the intestinal mucosa, are often confined within the lumen of the epithelial lining, although they can sometimes break through this physical barrier and cause infection. After entering the body, these commensal bacteria are taken up by resident macrophages and then transported by DCS to local lymphoid aggregates that drive the adaptive immune response. The human immune system has different abilities to defend against external pathogens in different parts of the female genital tract. There are differences between the lower genital tract (the vagina and vaginal part of the cervix) and the upper genital tract (the uterus and endocervix). Like the intestine, the vagina consists of a symbiotic flora in which the immune system must allow the growth of beneficial microorganisms while preventing the growth of pathogens. The human immune system has different capacities to defend against external pathogens in different parts of the female genital tract, with differences between the lower genital tract (vagina, vaginal part of the cervix) and the upper genital tract (uterus, endocervix). Like the intestine, the vagina consists of a symbiotic flora in which the immune system must allow the growth of beneficial microorganisms while preventing the growth of pathogens.

In summary, a good and stable human microbiome can play an important role in the local interaction between the embryo and the endometrium, and can influence implantation, placentation, and embryo growth, ultimately affecting the course and outcome of pregnancy.

## Data availability statement

The original contributions presented in the study are included in the article/supplementary material. Further inquiries can be directed to the corresponding authors.

## Ethics statement

All individuals involved in this study signed informed consent. The study was approved by Ethics Committee of Shanghai Tongji Hospital and in line with the ethical standards of the Declaration of Helsinki.

## Author contributions

The authors LS, LH, and AS conducted bibliographic research and authored the manuscript. WW, WH, CJ, and YY gathered patient data and conducted statistical analysis. All authors participated to and approved the final draft of the research.
